# The Medical Society of the County of Erie

**Published:** 1894-07

**Authors:** Franklin C. Gram

**Affiliations:** Secretary


### THE MEDICAL SOCIETY OF THE COUNTY OF ERIE.

Reported by FRANKLIN C. GRAM, M. D., Secretary.

The semi-annual meeting of the Medical Society of the County of Erie was called to order at 11 o'clock A. M., on Tuesday, June 12, 1894, by the President, Wm. H. Gail, M. D., at the rooms of the Buffalo Academy of Medicine.

In opening the proceedings the president stated that as he had not been present when elected to office he would take this opportunity to thank the members for the honor conferred on him. He then called for the reading of the minutes of the annual meeting held January 9th, and the special meeting held January 23, 1894. These were read and confirmed.

Dr. G. W. McPherson, of the committee on membership, reported favorably on the names of Drs. Ludwig Schroeter, William G. Taylor and Helene J. C. Kuhlman. They were unanimously elected members of the society. He also reported favorably upon the names of Drs. Wm. Meisburger, Maud J. Frye and Wm. C. Fritz, but stated that he had not been able to obtain the signatures of the remainder of the committee to the applications. On motion of Dr. Abbott these three applicants were also elected to membership, subject to the committee's completion of the necessary formality.

Applications for membership were received from Drs. N. Victoria Chappell, Albert H. Macbeth, G. B. Hepp, Charles A. Clements, Wm. G. Bissell and Arthur T. O'Hara. They were referred to the committee on membership.

Dr. W. C. Callanan moved for the appointment of a committee of three to draft a circular to be sent to all members, asking them to donate medical works to the society's library. The motion was adopted and the chair appointed Drs. Callanan, Eli Long and the Secretary.

Dr. John J. Walsh moved that this society endorse the bill relative to the office of coroner, now pending before the constitutional convention. Carried.

Papers were then read on The Purely Physical Examination of the Digestive Organs, by Dr. A. L. Benedict; Treatment of Neurasthenia, by Dr. Wm. C. Krauss; Clinical Experience with Smallpox During the Epidemic of 1888–9, by Dr. C. B. Le Van, ex-resident physician to the Buffalo Quarantine Hospital. Dr. McPherson also read an original poem.

A discussion followed, after which a vote of thanks was tendered to the essayists.

The secretary reported that he had received a communication from the Medical Society of the State of New York calling attention to the fact that the Medical Society of the County of Erie is now entitled to six delegates in the State society instead of five as formerly; also that the president of each county society is now an ex-officio member of the State society. He had also been requested to call the society's attention to the Merritt H. Cash prize for the best original essay on any medical or surgical subject. The conditions are that the competitor must be a member of a county medical society in this State, and the essay must be sent to the chairman of the committee, Dr. Franklin Townsend, 2 Park place, Albany, prior to January 1, 1895. The secretary reported that the by-laws and membership list of the society had been printed and a copy mailed to each member.

There were no reports from the board of censors or from the committee on hygiene.

The treasurer called attention to a resolution adopted at a previous meeting, by which all members who are three years in arrears with their dues will be dropped from the list.

Dr. Eli H. Long reminded the society that the Central New York Medical Society would meet in Buffalo next October, and that it will be necessary to appoint delegates and also to make arrangements to entertain the society. Dr. Bartlett stated that much valuable time had been lost at other places through formal dinners and thought that a luncheon would be better. Other members concurred in this opinion, and on motion of Dr. Krauss the sum of $50 was appropriated for this purpose. The chair appointed Drs. Krauss, Benedict and Eli Long as the committee to make the necessary arrangements.

The society then adjourned.

